# Supplementary material for: Effects of Coumarin on Rhizosphere Microbiome and Metabolome of Lolium multiflorum
Source: Plants (Basel). 2023 Mar 1;12(5):1096. doi: 10.3390/plants12051096 (PMC10005730; doi:10.3390/plants12051096)
Supplement: Supplementary file 1 [file plants-12-01096-s001.zip › plants-2235789-supplementary.pdf]

Supplementary file

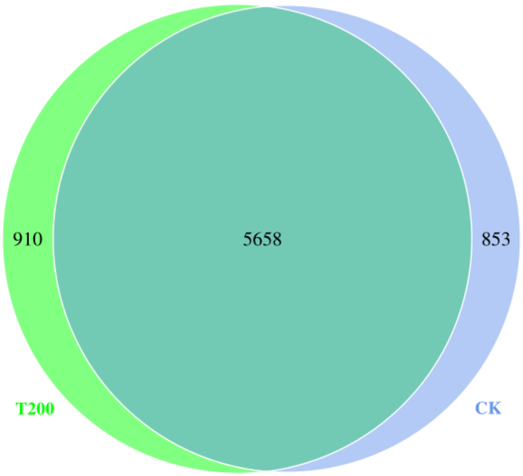

Figure S1. Shared bacterial OTUs between CK and T200 groups.

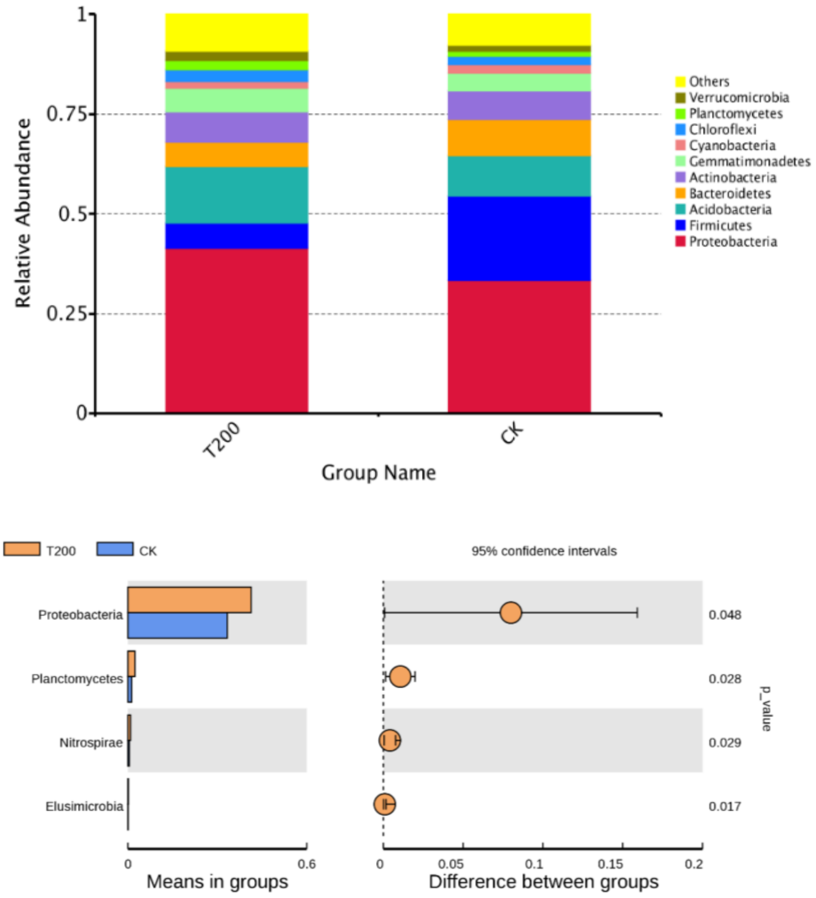

Figure S2. Relative abundance difference at phylum level among different treatments.The above figure is the main phyla detected in the samples.The left side of the figure below shows the relative abundance of the phylum with significant differences, and the right side of the figure below shows the significant differences of

the phylum with significant differences.

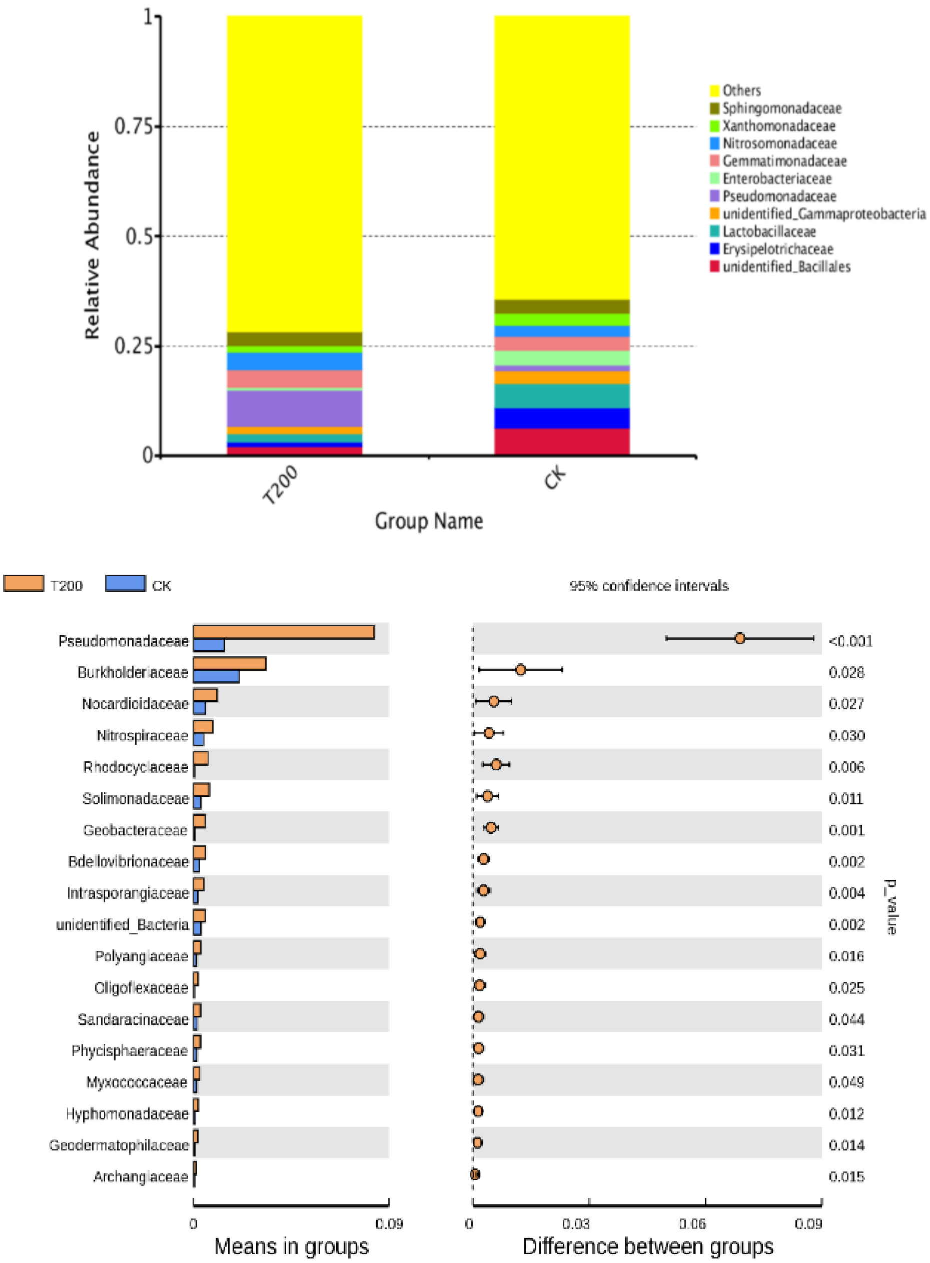

Figure S3. Difference of relative abundance in family level.The above figure is the main family detected in the samples.The left side of the figure below shows the relative abundance of the family with significant differences, and the right side of the figure below shows the significant differences of the family with significant differences.

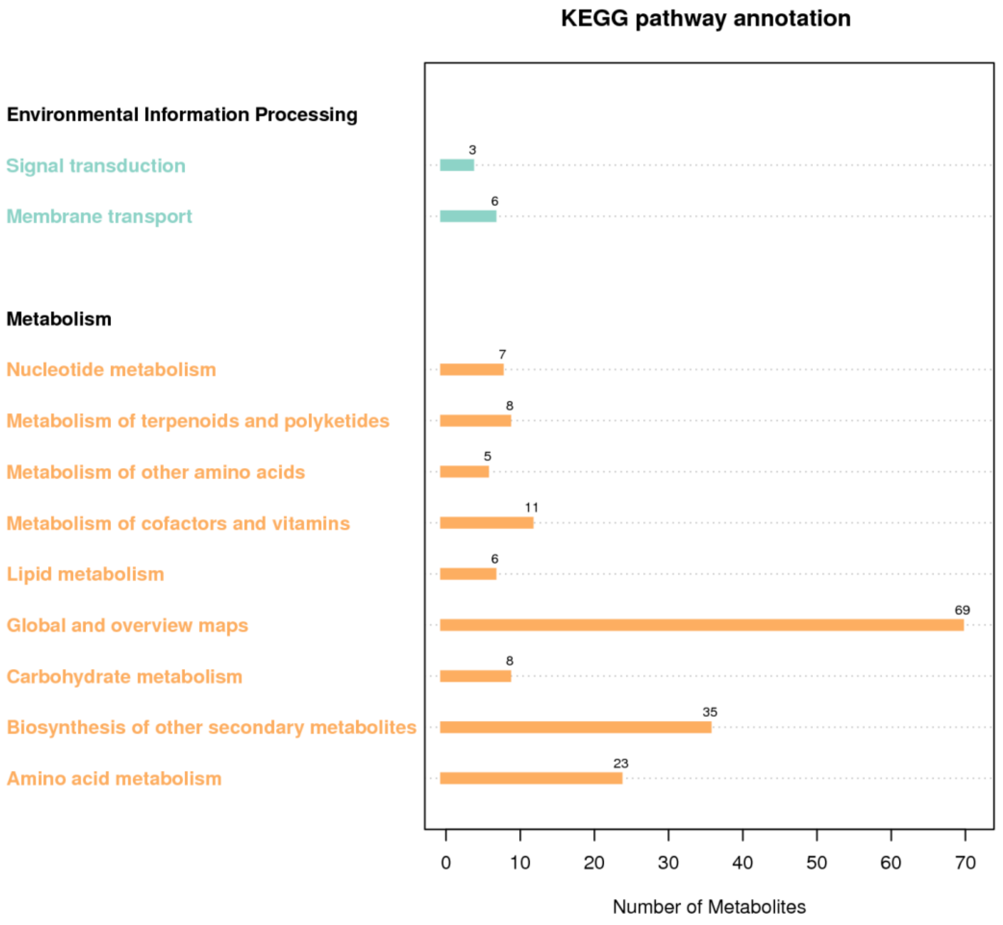

Figure S4. The only root non targeted metabolome and KEGG pathway annotation that can identify and annotate metabolites of KEGG pathway.
